# Detection of Conversion from Mild Cognitive Impairment to Alzheimer's Disease Using Longitudinal Brain MRI

**DOI:** 10.3389/fninf.2017.00016

**Published:** 2017-02-24

**Authors:** Zhuo Sun, Martijn van de Giessen, Boudewijn P. F. Lelieveldt, Marius Staring

**Affiliations:** ^1^Division of Image Processing, Department of Radiology, Leiden University Medical CenterLeiden, Netherlands; ^2^Department of Intelligent Systems, Delft University of TechnologyDelft, Netherlands

**Keywords:** Alzheimer's disease, Mild Cognitive Impairment (MCI), conversion, MRI, Stationary Velocity Field (SVF), non-rigid Registration, parallel Transport, SVM classification

## Abstract

Mild Cognitive Impairment (MCI) is an intermediate stage between healthy and Alzheimer's disease (AD). To enable early intervention it is important to identify the MCI subjects that will convert to AD in an early stage. In this paper, we provide a new method to distinguish between MCI patients that either convert to Alzheimer's Disease (MCIc) or remain stable (MCIs), using only longitudinal T1-weighted MRI. Currently, most longitudinal studies focus on volumetric comparison of a few anatomical structures, thereby ignoring more detailed development inside and outside those structures. In this study we propose to exploit the anatomical development within the entire brain, as found by a non-rigid registration approach. Specifically, this anatomical development is represented by the Stationary Velocity Field (SVF) from registration between the baseline and follow-up images. To make the SVFs comparable among subjects, we use the parallel transport method to align them in a common space. The normalized SVF together with derived features are then used to distinguish between MCIc and MCIs subjects. This novel feature space is reduced using a Kernel Principal Component Analysis method, and a linear support vector machine is used as a classifier. Extensive comparative experiments are performed to inspect the influence of several aspects of our method on classification performance, specifically the feature choice, the smoothing parameter in the registration and the use of dimensionality reduction. The optimal result from a 10-fold cross-validation using 36 month follow-up data shows competitive results: accuracy 92%, sensitivity 95%, specificity 90%, and AUC 94%. Based on the same dataset, the proposed approach outperforms two alternative ones that either depends on the baseline image only, or uses longitudinal information from larger brain areas. Good results were also obtained when scans at 6, 12, or 24 months were used for training the classifier. Besides the classification power, the proposed method can quantitatively compare brain regions that have a significant difference in development between the MCIc and MCIs groups.

## 1. Introduction

Alzheimer's disease (AD), one of the most common cases of dementia, is an age related degenerative brain disease. It is usually diagnosed in people over 65 years old (Alzheimer's Association, 2014). Brookmeyer et al. (2007) pointed out that the current number of AD patients worldwide will increase fourfold by the year 2050, from 26.6 million to above 100 million. Early detection and treatment is necessary to slow down the disease progress and decrease the societal cost of AD. If early treatment could delay the disease onset or slow down the disease progression by 1 year, this will yield almost nine million fewer AD patients in the world by 2050 (Brookmeyer et al., 2007). Different kinds of measurements and biomarkers have been used in early detection and prediction of AD, e.g., structural brain MRI (Frisoni et al., 2010), metabolic brain alterations measured by fluorodeoxyglucose positron emission tomography (FDG-PET) (De Santi et al., 2001), and pathological amyloid depositions measured from cerebrospinal fluid (CSF) (Leon et al., 2007; Mattsson et al., 2009). Among all these measurements, Magnetic Resonance Imaging (MRI) plays an increasingly important role in early detection of Alzheimer's disease because of its non-invasiveness, availability, and high sensitivity to change (Frisoni et al., 2010). Therefore, it is commonly used as part of the clinical assessment for the diagnosis of AD.

One of the key questions in Alzheimer's disease research is to understand the disease progression over a long period of time, where it is desirable to find the trend before manifestation of clinical symptoms. It is known that Mild Cognitive Impairment (MCI) is an intermediate stage between healthy and diseased, and possibly predicts the onset of Alzheimer's disease. However, not all MCI subjects develop AD. Based on whether the MCI patient will convert to AD during a study, the MCI group is typically divided into a stable group (MCIs) and a convertor group (MCIc). A meta-analysis (Mitchell and Shiri-Feshki, 2008) on 15 studies showed that the total number of patients who had progressed to dementia in studies lasting less than 5 years was 27.4%, while the total number of patients who had progressed to dementia by the end of the studies lasting up to 10 years was 31.4%. The conversion usually happens within the first 3 years after being diagnosed as MCI and the conversion rate drops dramatically in later years. Such a finding indicates that the MCIs group not simply takes longer to convert. Therefore, to early and sensitively detect AD, it is important to distinguish between cases that convert from MCI to AD and the cases that remain stable.

In the last 10 years, many cross-sectional structural MRI-based methods have been proposed to automatically distinguish between healthy controls (HC) and AD patients (Fan et al., 2005, 2007, 2008b; Davatzikos et al., 2008; Klöppel et al., 2008; Cuingnet et al., 2013), and some of them report a classification accuracy as high as 90%. Although such methods have diagnosed Alzheimer's disease, it is still too late for treatment, since most drugs approved by the U.S. Food and Drug Administration (FDA) are more likely to have a significant impact in the early stages of the disease (Crismon, 1994; Schneider et al., 2006; Kozauer and Katz, 2013).

Davatzikos et al. (2009, 2011) extended cross-sectional image-based classification to distinguish between MCIc and MCIs subjects and directly use healthy and AD brain images to train a classifier or regressor, and then use an AD-likeness score to distinguish between MCIc and MCIs (Davatzikos et al., 2009, 2011). These methods can minimize the empirical error that separates AD and HC subjects, but do not model the dynamic information of brain changes from healthy to AD. Therefore, such methods may fail to distinguish between MCIc and MCIs subjects, since disease progression over time may be more indicative than a static assessment at a fixed point in time.

Recently, methods were developed that learn a task-specific classifier to detect MCI convertors using cross-sectional datasets, which contain only one scan for each subject belonging to either the MCIc or the MCIs group (Chupin et al., 2009; Misra et al., 2009; Querbes et al., 2009; Cuingnet et al., 2011; Koikkalainen et al., 2011; Westman et al., 2011; Wolz et al., 2011; Cho et al., 2012; Eskildsen et al., 2013; Guerrero et al., 2014). Among these methods, different kinds of features are extracted from the image to train a discriminative model. Popular features include the hippocampus volume (Chupin et al., 2009), cortical thickness (Querbes et al., 2009; Westman et al., 2011; Wolz et al., 2011; Cho et al., 2012; Eskildsen et al., 2013), voxel-based morphometry (VBM) (Misra et al., 2009; Davatzikos et al., 2011) and tensor-based morphometry (TBM) (Koikkalainen et al., 2011; Wolz et al., 2011; Eskildsen et al., 2013). These methods also do not to take the dynamic information of brain changes into consideration. A second issue is the time at which the scan is taken, which is somewhat random and frequently in a later stage of the disease.

Since anatomical development in the brain is due to both normal aging as well as Alzheimer's disease progression (Lorenzi et al., 2012), and since there is individual variation in brain anatomy, it is hard to distinguish between MCIc and MCIs from a single scan. Also, AD can be treated as an accelerated aging process (Davatzikos et al., 2011), i.e., subjects with little AD-related and more age-related development may have similar anatomy as AD patients. Older subject are more similar to AD, and the subject's age at the baseline scan may introduce bias in the decision.

To avoid such scan time bias, some methods have started to include longitudinal image information. Zhang and Shen (2012) and Liu et al. (2013) use longitudinal structure-wise volumetric measurements as features for a sparse multi-task classifier. However, these methods summarize the anatomical development of the whole brain into a few scalar measurements and lose detailed anatomical information. Also, they do not model how the brain anatomy develops over time for the MCIc and MCIs groups. Similarly, Lorenzi et al. (2015a) summarizes the longitudinal change of each structure as a regional flux computed from the longitudinal deformation between the baseline and follow-up images. Fiot et al. (2012, 2014) directly use the longitudinal deformation of the hippocampus to distinguish between MCIs and MCIc, but without considering other brain structures.

In this study, we propose a new method that encodes the subject's longitudinal information by pairwise non-rigid registration between its baseline and follow-up images, and aligns this longitudinal information into a common space for classification. Our method can both distinguish between MCIc and MCIs subjects, as well as compute the mean developmental trajectory of the brain anatomy over time, for each group. In our approach, the development of the brain is represented by the anatomical correspondence between the baseline and follow-up scans from the same subject. In order to make the anatomy of different subjects comparable, we use the Schild's Ladder method (Lorenzi and Pennec, 2013) to transport the anatomical correspondence to a common template. A Support Vector Machine (SVM) classifier is learned to separate MCIc from MCIs. Our method can additionally be used for groupwise analysis, and compute the significant regions that develop differently between the MCIc and the MCIs groups. To investigate the influence of different parameters on the final result, we compare the effect of parallel transport, template selection, registration parameters and follow-up time on the final classification result. Our method thus features dense as well as longitudinal information, and can be used for classification as well as in studying groupwise differences.

The remainder of this paper is structured as follows: we first describe the dataset selection, image acquisition and preprocessing in Section 2. In Section 3, we present the proposed method in detail. The experiments and results are shown in Section 4. We discuss the results and compare the proposed method with state-of-the-art methods in Section 5. Finally, the conclusions are given in Section 6.

## 2. Materials

### 2.1. Subjects

All data used in this paper was obtained from the Alzheimer's Disease Neuroimaging Initiative (ADNI) (http://adni.loni.usc.edu). The ADNI was launched in 2003 by the National Institute on Aging (NIA), the National Institute of Biomedical Imaging and Bioengineering (NIBIB), the Food and Drug Administration (FDA), private pharmaceutical companies and non-profit organizations, as a $60 million, 5-year public-private partnership. The primary goal of ADNI has been to test whether serial MRI, PET, other biological markers, and clinical and neuropsychological assessment can be combined to measure the progression of MCI and early AD. Determination of sensitive and specific markers of very early AD progression is intended to aid researchers and clinicians to develop new treatments and monitor their effectiveness, as well as lessen the time and cost of clinical trials.

The principal investigator of this initiative is Michael W. Weiner, MD, VA Medical Center and University of California, San Francisco. ADNI is the result of efforts of many co-investigators from a broad range of academic institutions and private corporations, and subjects have been recruited from over 50 sites across the U.S. and Canada. The initial goal of ADNI was to recruit 800 subjects but ADNI has been followed by ADNI-GO and ADNI-2.

We select and define MCI subjects according to the label that is available for each subject for most of the visits: MCI, MCI to dementia, or dementia. According to the ADNIMerge file (https://adni.loni.usc.edu/wp-content/uploads/2012/08/instruction-ADNIMERGE-packages.pdf)^1^, there are 837 subjects available that have either the label “MCI” or the label “MCI to dementia” at baseline. From those subjects we select only those that have a baseline scan, together with follow-up scans at 6, 12, 24, and 36 months. We exclude subjects that have a non-monotone diagnosis, for example going from MCI to dementia to MCI again, resulting in 143 remaining subjects. We further exclude subjects with only “MCI” or “MCI to dementia” labels where the last visit has no diagnosis available, as these subjects are considered unclear to belong to either the MCIs or the MCIc group. This finally results in 110 remaining MCI subjects. A subject is defined as stable if no visit is labeled as dementia, and as a converter if the label dementia is present. We then have 43 MCIs and 67 MCIc subjects.

The demographic characteristics of the selected study population are summarized in Table 1. The diagnosis of each visit is summarized in Table 2. We can see that some MCIc subjects convert to AD later than 36 months. Although the maximum considered time interval in our study is 36 months, we still treat such subjects as MCIc. We can also see that no subject converted to AD in the first 6 months after the baseline visit.

**Table 1:** **Demographic characteristics of the studied longitudinal dataset (from ADNI)**.

**Group**	**Number**	**Baseline age**	**Gender**	**Baseline MMSE^a^**	**Follow-up MMSE**
MCIc	67	74.4±6.9 [55.1−87.7]	43M / 24F	26.6±1.7 [24−30]	22.4±4.5 [10−30]
MCIs	43	75.5±6.8 [57.8−86.4]	35M / 8F	27.7±1.9 [24−30]	27.9±2.1 [22−30]

a *MMSE means Mini-Mental State Examination*.

**Table 2:** **Diagnosis distribution at each visit and MMSE of each group at each visit (in term of months)**.

	**Baseline**	**6**	**12**	**24**	**36**	**Last diagnosed visit**
MCI	110	104	91	63	56	43
MCI to dementia	0	6	13	13	8	0
Dementia	0	0	6	34	46	67
MCIs MMSE	27.7±1.9	27.9±2.1	28.2±1.8	27.7±2.3	28.0±2.1	
MCIc MMSE	26.6±1.7	25.4±2.4	25.6±2.4	23.6±4.0	22.4±4.5	

### 2.2. Acquisition and preprocessing

All selected scans are T1-weighted 1.5T MR images acquired with machines from Philips, Siemens or GE. Acquisitions were made in different clinical centers across North America according to the ADNI acquisition protocol (http://adni.loni.usc.edu/methods/mri-analysis/mri-acquisition/). To enhance the standardization among scans acquired from different clinical sites and platforms, pre-processing and post-precessing is made to correct certain image artifacts (Jack et al., 2008). In our study, all the images downloaded from ADNI are from the MP-RAGE sequence and pre-processed by ADNI. In the ADNI pre-processing and image correction pipeline, images from the Philips machine are intensity corrected by the N3 method (Sled et al., 1998), while images from Siemens or GE machines are grad-warped, followed by B1 bias field correction and N3 intensity non-uniformity correction. All images were preprocessed through the same fully automatic pipeline, as described by Coupé et al. (2015). Inside this pipeline, the inhomogeneities are corrected by N3, the brain is extracted by BEaST (Eskildsen et al., 2012), and the intensity is linearly normalized to the MNI template intensity. After preprocessing each image independently using these identical steps, all follow-up images are registered to its baseline image using a similarity transform (rigid plus isotropic scaling) with elastix (Klein et al., 2010).

## 3. Methods

**Figure 1:**
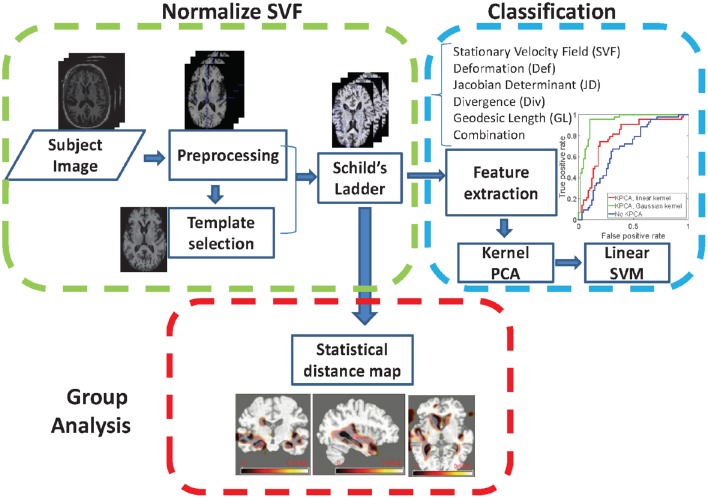
**Illustration of the proposed pipeline for longitudinal brain development analysis**.

In this section, we present the proposed method and briefly describe how to perform a group analysis on the MCIc and MCIs groups. The proposed pipeline is summarized in Figure 1. First, see Section 3.1, we describe how to represent the anatomical development between the baseline and follow-up images using LogDemons non-rigid registration (Vercauteren et al., 2009), and how to normalize the anatomical development in a common template space by building a Schild's Ladder on the image manifold. Second, see Section 3.2, we describe the features that describe the anatomical development, a dimensionality reduction step that uses kernel principle component analysis (KPCA), and the Support Vector Machine (SVM) classification method. Finally, group-wise analysis between the MCIc and the MCIs group is detailed in Section 3.3.

### 3.1. Normalization of anatomical development

#### 3.1.1. Symmetric LogDemons registration

In this work, the symmetric LogDemons method (Vercauteren et al., 2009) is used to compute non-rigid diffeomorphic transformations between baseline and follow-up scans to estimate brain development, and also between different subjects for normalization by parallel transport. Originating from the Demons method (Thirion, 1998), symmetric LogDemons uses the stationary velocity field (SVF) *v* to parameterize the diffeomorphic deformation φ by the exponential map φ = *Exp*(*v*), which is used to align the moving image *F* with the fixed image *M*. The following cost function is optimized:

(1) $$\begin{array}{l} v^{*}=\underset{v}{\text{argmin}}\|(F-M◦Exp(v)){\|}_{2}\\ \;+\|(M-F◦Exp(-v)){\|}_{2}+Reg(v;\sigma ), \end{array}$$

where the first two terms measure the dissimilarity of *F* and *M* after forward and backward deformation, and the third (*Reg*(*v*; σ)) regularizes the SVF by smoothing it with a Gaussian kernel with standard deviation σ. The resulting stationary velocity v^*^ is the optimal solution that minimizes the above cost function. According to Cachier et al. (2003), this problem can be decomposed into an alternative minimization problem, for which a more efficient computation is proposed in (Vercauteren et al., 2009). As proven in Lorenzi and Pennec (2013), the deformation trajectory can be represented as a one-parameter geodesic path, controlled by a virtual time parameter *t*:

(2) $$\varphi_{t}(v)=Exp(v,t)=Exp(v\times t).$$

For *t* = 1 we obtain the deformation between fixed and moving image.

#### 3.1.2. Parallel transport by schild's ladder

**Figure 2:**
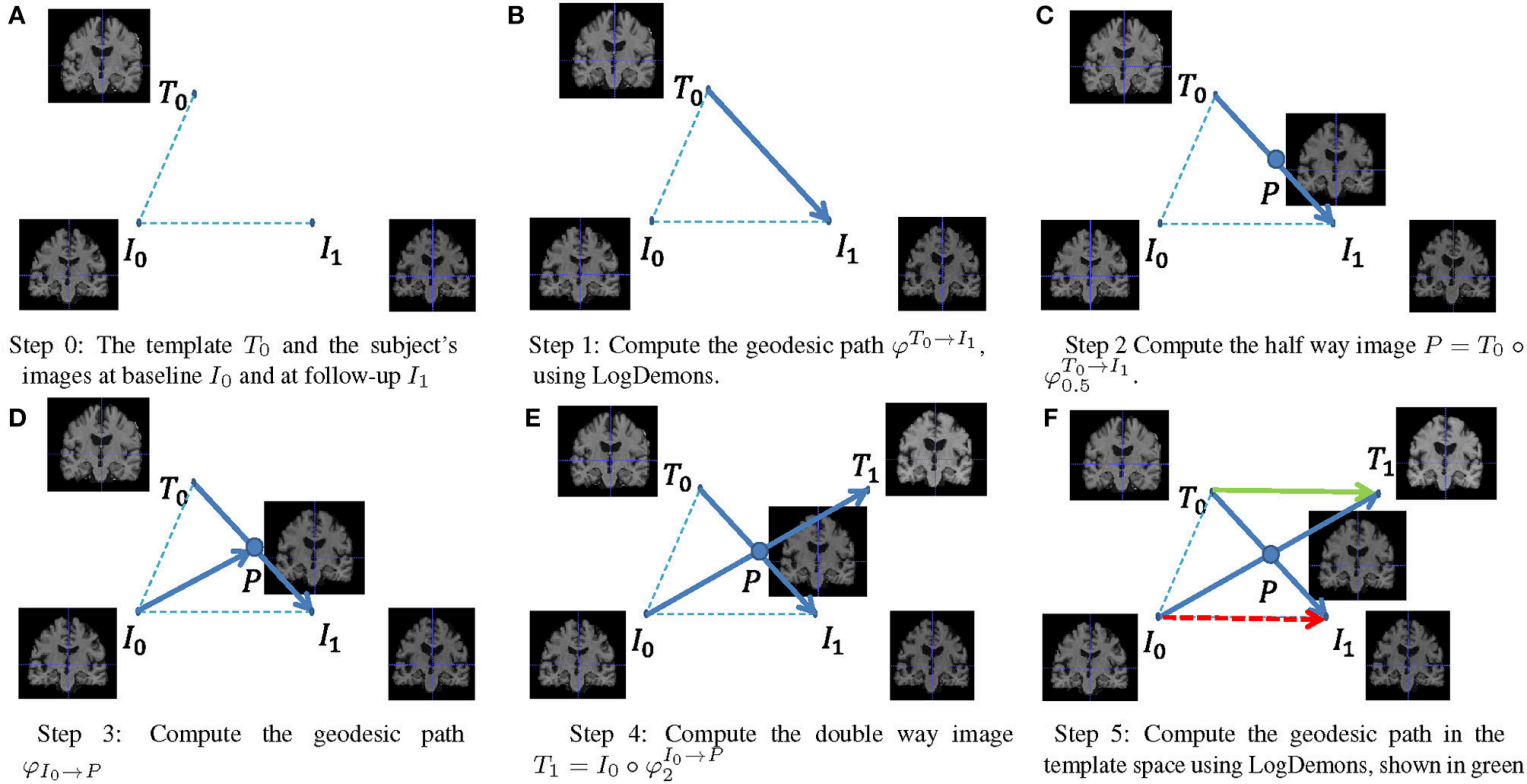
**Using Schild's Ladder to transport the SVF from subject space to a common reference space**.

It is not possible to directly compare SVFs from different subjects, since they have different coordinate systems. It is therefore necessary to normalize the SVFs to a common template space, for which we employ the parallel transport method (do Carmo, 1992). In our approach, we use the Schild's Ladder parallel transport method (Lorenzi and Pennec, 2013) to deform the subject space follow-up image *I*_1_ to its corresponding image *T*_1_ in the template space, and compute the normalized SVF by symmetric LogDemons registration from *T*_0_ to *T*_1_. The Schild's Ladder method is summarized by the following steps and illustrated in Figure 2.

1. Compute the diffeomorphic deformation $\varphi^{T_{0}\to I_{1}}$ to deform the template image *T*_0_ to the follow-up image *I*_1_ in the subject space, and the corresponding SVF $v^{T_{0}\to I_{1}}$.
2. Define the half-space image $P=T_{0}◦\varphi_{0.5}^{T_{0}\to I_{1}}$.
3. Compute the diffeomorphic deformation $\varphi^{I_{0}\to P}$ that deforms the subject space baseline image *I*_0_ to image *P*.
4. Define the template space follow-up image *T*_1_ as the double deformed subject baseline image as $T_{1}=I_{0}◦\varphi_{2}^{I_{0}\to P}$.
5. Compute the SVF *v* that deforms *T*_0_ to *T*_1_, which transports the subject's anatomical correspondence into the template space.

Lorenzi and Pennec (2013) proposed an alternative implementation of Schild's Ladder using the Baker-Campbell-Hausdorf (BCH) approximation to work directly on the velocity field. In this implementation, different subjects tend to have different SVF amplitude, need a different number of BCH approximation steps and possibly use multiple ladders. In this work, We use a computationally attractive approach that omits the BCH approximation and requires only a single ladder per normalization. However, the method proposed by Lorenzi and Pennec (2013) could also be used in the proposed framework. In our approach, the Schild's Ladder is applied to each subject's baseline and follow-up image pair independently.

#### 3.1.3. MCI template selection

**Figure 3:**
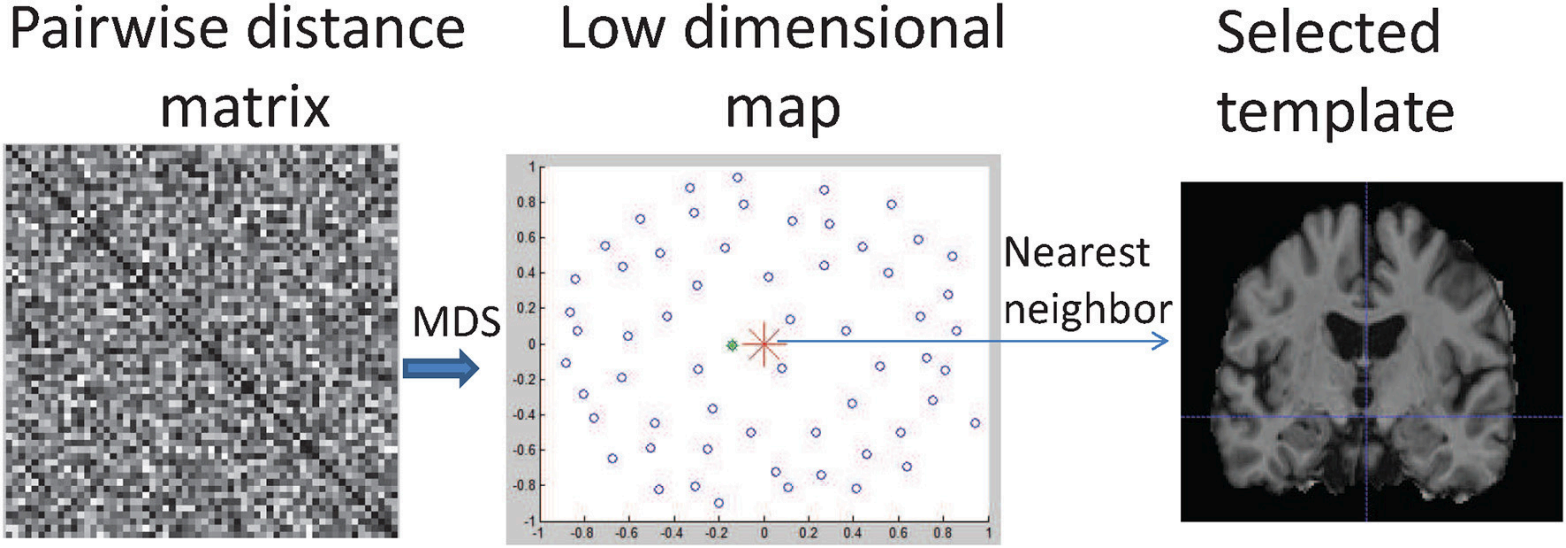
**Illustration of MDS-based template selection**. Step 1: Compute a matrix containing the pairwise distances between globally aligned baseline images; 2: Map images into a low dimensional space (shown as blue circles) using MDS; 3: Find the image (shown as the green dot) closest to the mean point (shown as red star) in the low dimensional map.

In our study, a template image is needed for the transportation of the SVFs. The template needs to be selected in an adaptive and unbiased way to fit the study population and minimize the registration error. In our approach, we use the multidimensional scaling method to select the least biased template from the baseline images of our study population, according to Park et al. (2005), as shown in Figure 3. The selected image is an approximation of the group mean image in the given image manifold.

To learn the mean of a population, we first compute the pairwise geodesic distances between two images *i* and *j* as $d_{i,j}=|v_{i,j}{|}_{2}^{2}=\underset{q\in I}{\sum }\langle v_{i,j}\left(q\right),v_{i,j}\left(q\right)\rangle$, where *v*_*i, j*_ is the SVF that aligns these two images and 〈·, ·〉 denotes the inner product. The multidimensional scaling (MDS) method is used to map each high-dimensional geodesic distance on the image manifold into a 2D Euclidean space. The optimal MCI template image is then selected as the nearest neighbor of the arithmetic average in the 2D space, shown as the green dot in Figure 3. Compared to other commonly used unbiased template construction methods (Joshi et al., 2004; Lorenzen et al., 2005), this method is easy to implement and very suitable for parallel computing. To investigate the influence of a pre-defined general population template and a study-specific template, in the experiments we compare the MDS-based template with the commonly used MNI template.

### 3.2. Classification

In our study, we use the linear support vector machine (SVM) as the classification method. Given the training set {**f**_*i*_, *y*_*i*_}, where **f**_*i*_ is the feature vector and *y*_*i*_ is the label of subject *i* (MCIc or MCIs), we compute the optimal linear SVM model {**w**, *b*} by minimizing the cost function:

(3) $$\{w,b\}=\underset{w,b}{\text{argmin}}\|w{\|}_{2}^{2}+c\sum_{i\;=\;1}^{N}\max(0,1-y_{i}(\langle w,f_{i}\rangle +b)),$$

where {**w**, *b*} are the learned parameters of the linear model. The feature(s) used in the SVM could be the normalized SVF itself (shown as the green arrow in Figure 2), or features derived from it, or a combination of them. In this paper, we only use the SVF of a single time interval, i.e., from baseline to one of the follow-up images. We consider the following additional features:

- Jacobian Determinant (JD): defines the local volume ratio before and after registration. If it is larger than 1, the local volume increases and vice versa.
- Divergence (Div): locally defines how much volume flows in (Div > 0) or out (Div < 0).
- Geodesic Length (GL): defines the path length that a particle travels in the deformation trajectory.
- Deformation (Def): the deformation field generated from the normalized SVF using the exponential map ϕ = *Exp*(*v*).
- Combination of features: concatenate all these features into a longer feature vector.

Note that instead of the normalized SVF (green arrow in Figure 2) we could have also used the standard SVF that does not employ Schild's ladder (red arrow in Figure 2) as a distinguishing feature. We will compare the two in the experiments.

For our particular application the feature dimensionality of the original feature ${\text{g}}_{i}\in {\mathbb{R}}^{D}$ described above for each subject *i* is much larger than the number of training samples *N*, i.e., the number of subjects. To reduce the effect of overfitting on the classifier, we therefore employ dimensionality reduction on the original feature vector ${\text{g}}_{i}\in {\mathbb{R}}^{D}$ by mapping it to a space of reduced dimension thereby obtaining a new feature vector ${\text{f}}_{i}\in {\mathbb{R}}^{d},d\ll D$. For each subject *i*, the original feature ${\text{g}}_{i}\in {\mathbb{R}}^{D}$ is first z-score normalized, after which it is downsampled with a factor of 4 in each direction using nearest neighbor interpolation, and only voxels within the brain mask are considered. This yields features ${\text{h}}_{i}\in {\mathbb{R}}^{D_{2}}$. Since all features are based on the normalized SVF, they are aligned to the template space, and there is therefore no need to normalize them again as for example used in VBM. Secondly, we use a dimensionality reduction method based on Principal Component Analysis (PCA) for its simplicity, thereby mapping the downsampled feature **h**_*i*_ to a low dimensional space ${\text{f}}_{i}\in {\mathbb{R}}^{d}$, *d* ≪ *D*_2_. As the standard PCA approach is computationally unattractive for large feature sizes, we use the Kernel PCA (KPCA) approach (Schölkopf et al., 1997). For KPCA the kernel *K*(*i, j*) represents the pairwise relation between subjects *i* and *j* in the high dimensional space ${\mathbb{R}}^{D_{2}}$. For the linear case, the kernel is chosen as the inner product between two feature vectors **h**_*i*_ and **h**_*j*_: *K*(*i, j*) = 〈**h**_*i*_, **h**_*j*_〉. For the Gaussian case, $K\left(i,j\right)=exp\left(-\frac{{\left({\text{h}}_{j}-{\text{h}}_{i}\right)}^{2}}{2\theta^{2}}\right)$, where θ is the standard deviation of the Gaussian kernel. A standard PCA is then applied to the kernel *K* to obtain a low-dimensional representation **f**_*i*_, by projecting the kernel representation *K*(*i*, :) = {*K*(*i*, 1), ⋯ , *K*(*i, N*)} ∈ ℝ^*N*^ to the computed eigen vectors. This low-dimensional representation **f**_*i*_ is then used in the SVM, see Equation (3). We test the with linear as well as the Gaussian kernel in the experiments below.

### 3.3. Group analysis

Given a set of normalized SVFs from two clinical groups, we can find the regions that differ in anatomical development, by computing the voxelwise two-sample Hotelling's T-square test. To compute the Hotelling's T-square test, we treat SVFs from the two different groups (MCIc and MCIs) as two vector distributions. For each voxel position, we have two matrices of size *n*_1_ × 3 and *n*_2_ × 3, where *n*_1_ and *n*_2_ are the number of subjects in the MCIc and MCIs group, respectively. Each row in the matrix stores the SVF at this position, which is also one element in the vector distribution.

## 4. Experiments and results

In this section, we perform several experiments to investigate the influence of different aspects of our method on the classification results and compare the MCIc and MCIs groups. First, we experimentally validate different SVF smoothing strengths σ for the LogDemons registration. Second, we compare the performance of the different features, with and without dimensionality reduction. Third, we assess the effect of parallel transport on the classification. Fourth, the proposed MDS-based template is compared with the well-known MNI template, and the result shows that a study-specific template performs much better than a pre-defined general population template. Except for this experiment, all experiments use the MDS-based template. To prove the importance of both longitudinal and dense information as feature characteristics, we compare our results with two alternative type of features: the gray matter density map at baseline from (Klöppel et al., 2008) and the regional flux feature proposed by Lorenzi et al. (2015a). In our re-implementation these two methods are not exactly the same as the original ones, we only use the proposed features. The same linear SVM classification method and the same dataset are used. We also investigate the influence of follow-up time by using different follow-up times (6, 12, 24, and 36 months). We compute a statistical distance map between the MCIc and MCIs group. This map highlights the regions that develop differently between the MCIc and the MCIs groups.

In the experiment's default setting, for each subject, the follow-up image is first rigidly registered to the baseline image, and then the subject space is globally aligned to the template space using affine registration, both using elastix (Klein et al., 2010). For the LogDemons registration method, we use a multi-resolution approach with the default number of iterations (15 iterations in the first resolution, 10 iterations in the second resolution and 5 iterations in the highest resolution) and default second order BCH approximation (Hernandez et al., 2009; Vercauteren et al., 2009). For KPCA using the Gaussian kernel we use a standard deviation equal to 1.

The liblinear toolbox (Fan et al., 2008a) is used for linear SVM classification. The parameter *c* used in the SVM classification is chosen by a grid search over the discrete set {10^−3^, 10^−2^, 10^−1^, 0.5, 1, 2, 5, 10, 50, 10^2^, 10^3^}.

We use 10-fold cross-validation to test the performance of the proposed classification method, and use all the images for group analysis. In our classification experiment, we use accuracy (ACC), sensitivity (SEN), specificity (SPE), F1 score and area under the ROC curve (AUC) to measure the performance of the classification.

### 4.1. Smoothing parameter in LogDemons

**Figure 4:**
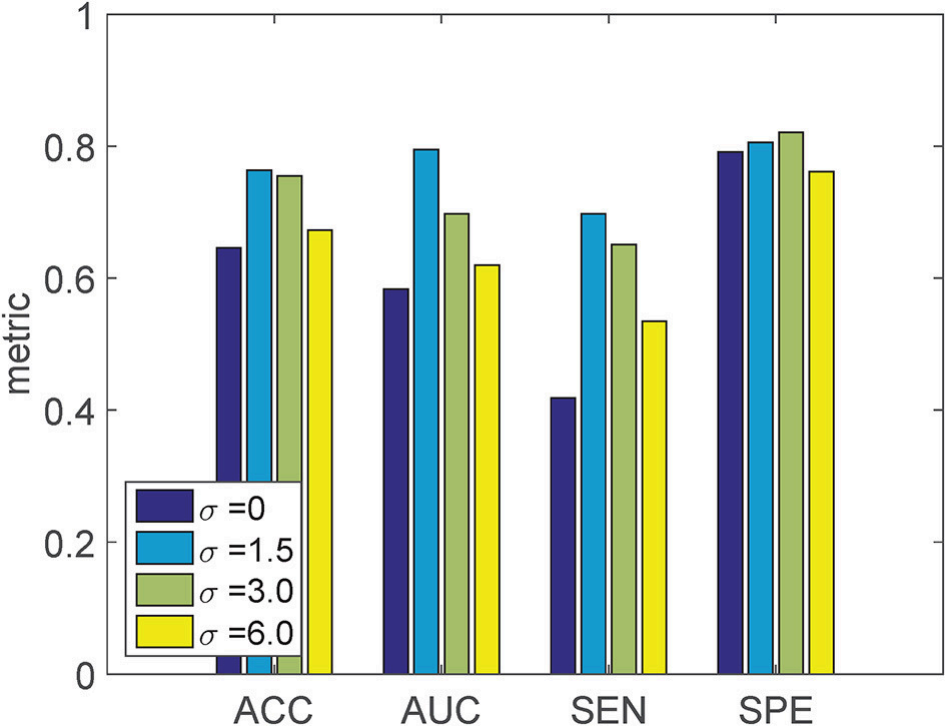
**Classification result of the proposed method using the SVF feature with varying SVF smoothing parameter σ**. KPCA with a linear kernel is used, employing the MDS-based template.

Since the LogDemons registration quality is influenced by the smoothing parameter σ, we inspect the influence of σ on the final classification accuracy using σ ∈ {0, 1.5 (default), 3, 6}. For each σ, we recompute Schild's Ladder, and train the linear SVM classifier on the SVF feature with linear KPCA dimensionality reduction. The result of the 10-fold cross-validation with different σ is shown in Figure 4.

We can see that when σ = 0, both the ACC and AUC is lower than the other settings of σ. As σ increases from 1.5 to 6, both ACC and AUC decrease, but are still better than σ = 0. The setting σ = 1.5 gives the best classification performance, and is used in further experiments.

**Figure 5:**
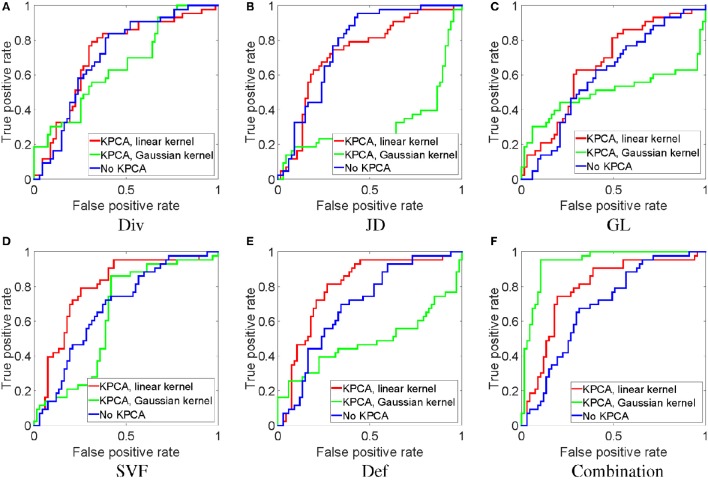
**ROC analysis of classification with different features with and without dimensionality reduction**. All experiments in this figure use the MDS-based template.

In this experiment, we perform classification based on the SVF feature itself, the derived features (JD, GL, Div, Def) and the combination of all the features. For each feature, we test KPCA with the linear as well as the Gaussian kernel. The resulting ROC curves are shown in Figure 5. We can see that for each single feature, in general the linear kernel performs better than the Gaussian kernel, and also is better than the original feature without KPCA. For the combination of all features, the Gaussian kernel performs substantially better than the linear kernel and the original feature without KPCA. While for single features the independence assumption of a linear kernel may still approximately hold, for the combination of related features (all derived from the SVF) this does not hold anymore. In that case the Gaussian kernel may be more appropriate, and indeed performs better. It can also be observed that the SVF and the deformation are the best performing single features, and that the combination of all features, using the Gaussian kernel, performs best overall.

### 4.2. With and without parallel transport

**Figure 6:**
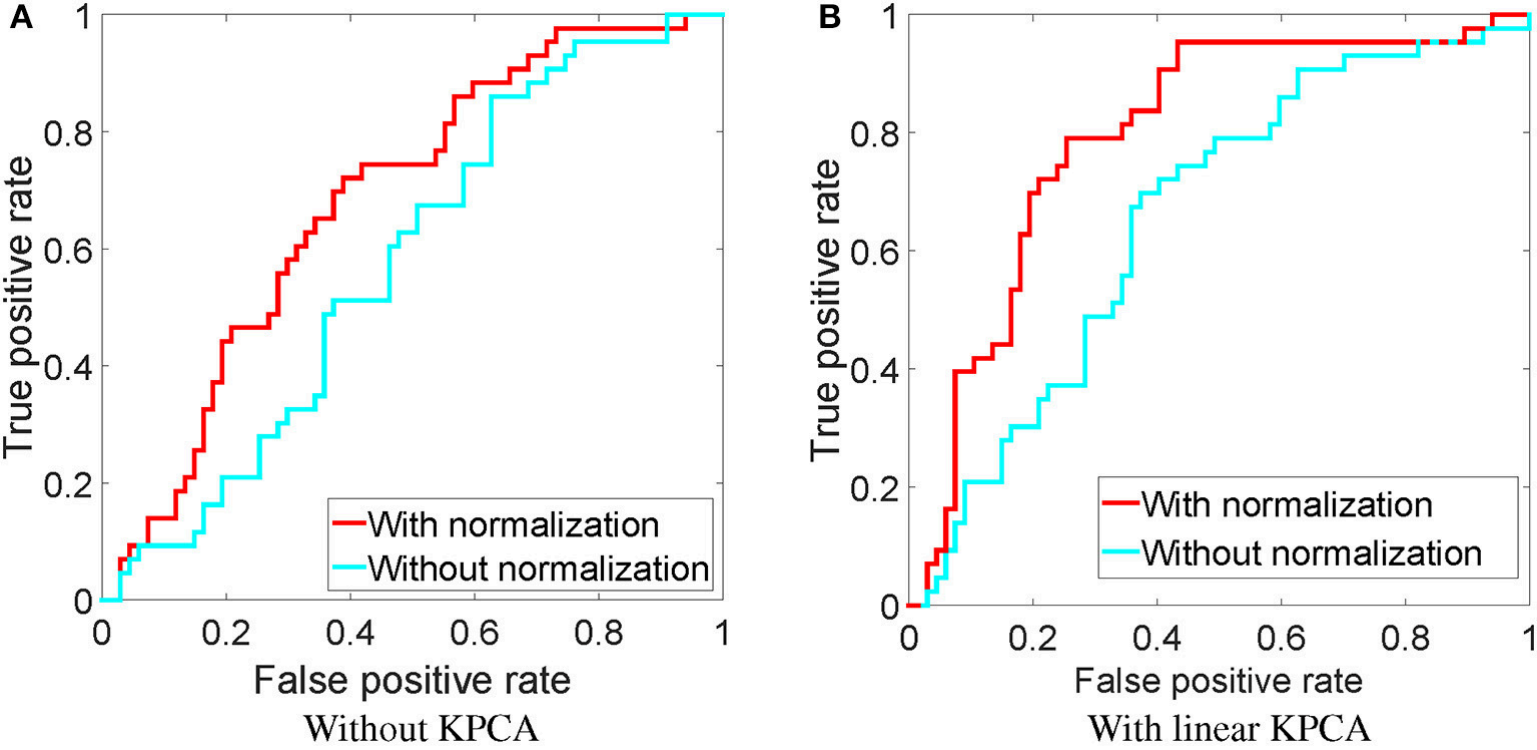
**ROC of classification using the downsampled SVF feature with and without normalization, using the MDS-based template**. **(A)** Without KPCA, **(B)** with a linear KPCA.

As discussed before, parallel transport is a necessary step to align the subject's features into a common space. Here, we compare the SVF feature with and without normalization by parallel transport, shown as the green and red arrows in Figure 2. We show the effect of normalization both on the original features, as well as on the dimensionality reduced version by KPCA using a linear kernel (this was the best kernel for SVF as a single feature, see Figure 5). The resulting ROC curves are shown in Figure 6. From this figure it is clear that the normalization by parallel transport is a necessary step for both theoretical and performance benefits.

### 4.3. Influence of template space

**Figure 7:**
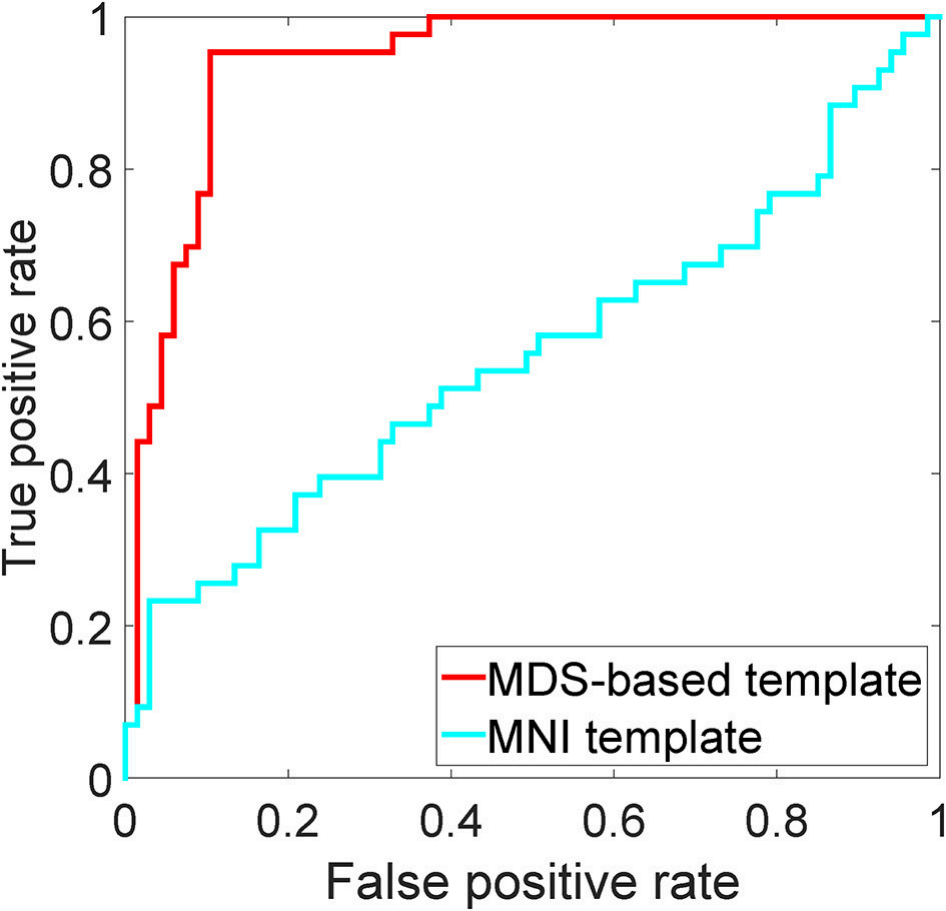
**ROC analysis of the MDS-based template and the MNI152 template using the combination of all features and KPCA with the Gaussian kernel**.

Although the template space selection is not the main goal of this work, we briefly compare the template image selected by the MDS method, see Section 3.1.3, with the MNI152 template (Fonov et al., 2009). For both templates, we use the same LogDemons registration parameters (σ = 1.5), and perform classification using the combination of all features and the Gaussian kernel for dimensionality reduction. The resulting ROC curve is shown in Figure 7.

### 4.4. Comparison with alternative methods

**Figure 8:**
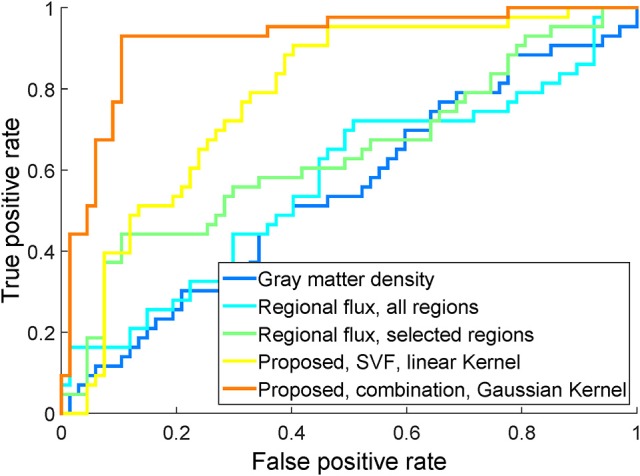
**ROC of classification using the proposed SVF feature (using linear KPCA), the proposed combination of features (using Gaussian KPCA), the structural longitudinal feature using all brain regions, the structural longitudinal feature using a subset of brain regions, and the dense cross-sectional feature, on the same study population**.

To compare with other methods fairly, we use the same linear SVM classification on the same dataset. For this experiment, we re-implement the dense gray matter density feature computed from the baseline image (Klöppel et al., 2008) and the sparse regional flux feature (Lorenzi et al., 2015b) as alternative methods. Our re-implementation is slightly different from the original paper. For the gray matter density feature, we computed the voxel-based morphometry (VBM) feature using the SPM8 toolbox (http://www.fil.ion.ucl.ac.uk/spm/software/spm8/), while in Klöppel et al. (2008) the authors use SPM5. For the sparse regional flux feature, we used structural flux, which is also used in Lorenzi et al. (2015b) and computed as the sum of divergences inside brain regions. Like Lorenzi et al. (2015b), we use the Automated Anatomical Labeling (AAL) segmentation (Tzourio-Mazoyer et al., 2002) by mapping it into the MDS-based template space. We use structural flux computed on all brain regions in the ALL segmentation, as well as computed only on selected brain regions (hippocampi, medial temporal lobes, posterior cingulate, and ventricles) as in Lorenzi et al. (2015b). For each of these approaches, we report the classification result in Figure 8. We can see that the proposed methods substantially outperform the others.

### 4.5. Comparing follow-up time

**Figure 9:**
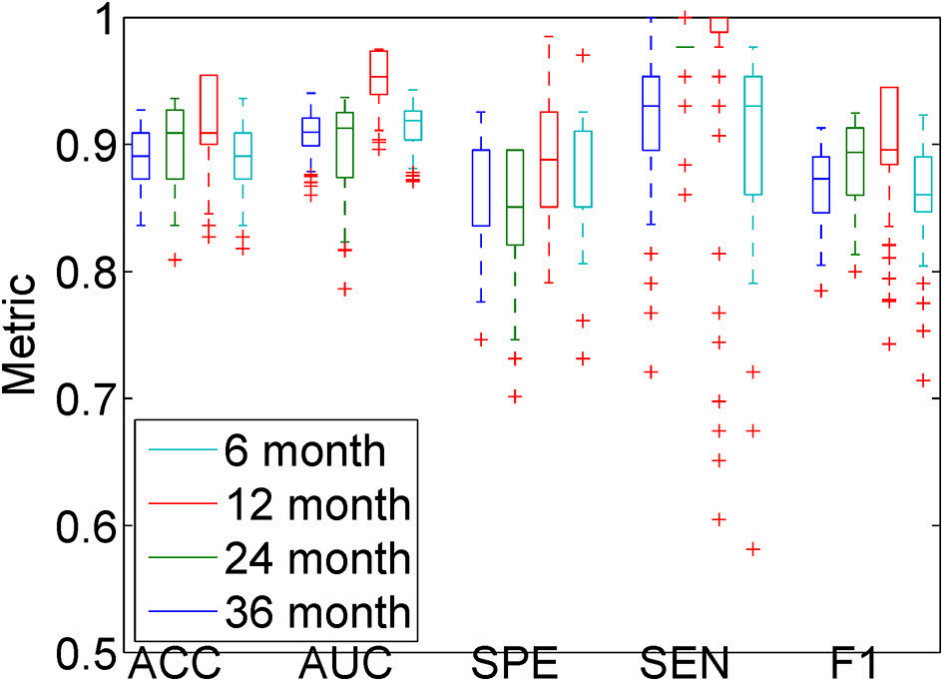
**Classification results for the different follow-up images, using the combination of all features and KPCA with a Gaussian kernel**. All experiments in this figure are employing the MDS-based template.

All the above results are based on the 36 month follow-up images. The same approach can be used on shorter time intervals. We performed an experiment on the 6, 12, 24, and 36 month follow-up images, using the combination of all features with Gaussian kernels for KPCA. Classification was repeated 500 times, each time with a different randomization in a 10-fold cross-validation. From Figure 9, it can be appreciated that when using a Gaussian kernel, the accuracy does not change a lot with different time intervals, and even the 6 month follow-up image can give a high accuracy and AUC. We performed Wilcoxon signed-rank tests, comparing the results of the different time intervals with those of the 36 month interval. The results are reported in Table 3. We can see that similar classification performance can be obtained with each time interval, but 12 and 24 month follow-up give slightly better classification performance than 6 month follow-up. The 36 month follow-up seems slightly worse, which may be attributed to an increase in registration difficulty for larger anatomical differences.

**Table 3:** **Comparing different follow-up times, using the combination of all features and Gaussian kernel KPCA**.

		**6 months**	**12 months**	**24 months**	**36 months**
ACC	Mean (Q1,Q3)	0.89 (0.87,0.91)	0.90 (0.87,0.93)	0.92 (0.90,0.95)	0.89 (0.87,0.91)
	*p*-value	0.3955	0.0225	0.0000	−
AUC	Mean (Q1,Q3)	0.91 (0.90,0.92)	0.90 (0.87,0.93)	0.95 (0.94,0.97)	0.91 (0.90,0.93)
	*p*-value	0.0335	0.0027	0.0000	−
SEN	Mean (Q1,Q3)	0.92 (0.90,0.95)	0.98 (0.98,0.98)	0.97 (0.99,1.00)	0.90 (0.86,0.95)
	*p*-value	0.0144	0.0000	0.0000	−
SPE	Mean (Q1,Q3)	0.87 (0.84,0.90)	0.85 (0.82,0.90)	0.89 (0.85,0.93)	0.88 (0.85,0.91)
	*p*-value	0.1685	0.0000	0.5500	−
F1	Mean (Q1,Q3)	0.87 (0.85,0.89)	0.88 (0.86,0.91)	0.90 (0.88,0.95)	0.86 (0.85,0.89)
	*p*-value	0.1886	0.0001	0.0000	−

*Here, we report the mean, first quartile (Q1) and third quartile (Q3) of the results of a 10-fold cross-validation, together with p-values of a Wilcoxon signed-rank test comparing shorter time intervals with the 36 months time interval*.

### 4.6. Group analysis

**Figure 10:**
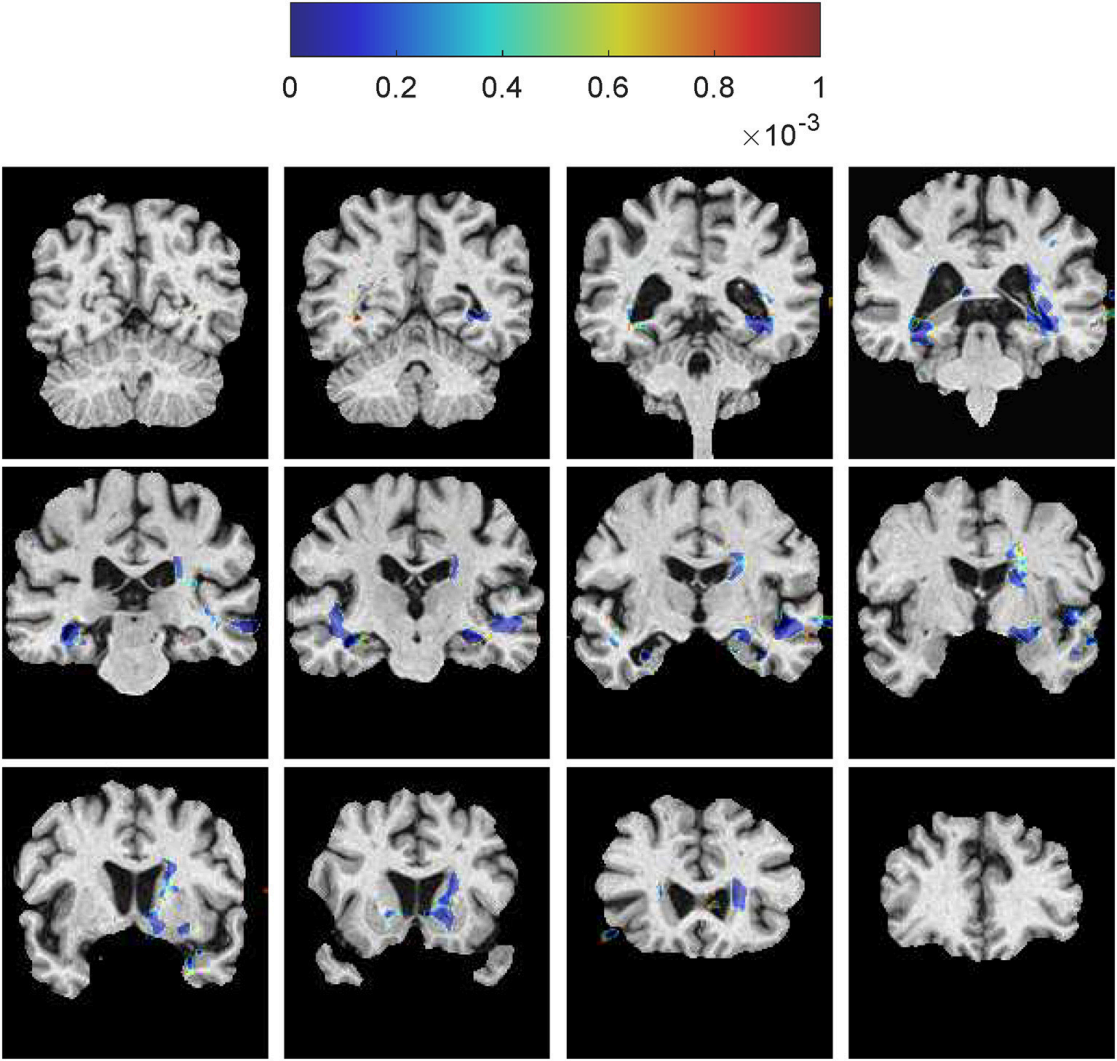
**Voxelwise two-sample Hotelling's T-square test on the SVFs between the MCIc and the MCIs groups**. The corpus callosum and the hippocampus show significant differences between these two groups. The color spots just outside the brain region are caused by smoothing of the SVFs during the LogDemons registration.

Based on the aligned SVFs of all subjects, see Section 3.1.2, we can do a group analysis to show the differences in development between the MCIc and MCIs groups. Before the group testing, we first performed a voxelwise Shapiro-Wilk test inside each group for each voxel in the brain mask, 82.2% voxels in MCIc group and 74.1% for the MCIs group fit the normal distribution. Then, we compute a voxelwise two-sample Hotelling's T-square test on the SVFs of the MCIc and the MCIs groups. Finally, we use the FDR method (Benjamini and Yekutieli, 2001) to correct the voxelwise independent *p*-value map for multiple comparison testing. The resulting FDR corrected *p*-value map (*p* ≤ 0.001) is shown in Figure 10. The corpus callosum and hippocampus show significant differences (*p* ≤ 0.001), which are confirmed by other MCI conversion studies (Wang et al., 2006; Zhang et al., 2013; Elahi et al., 2015).

### 4.7. Summary of all the results

The experimental results on the 36 month follow-up data are summarized in Table 4, for different features, KPCA kernels and LogDemons smoothing parameters. Note that here we performed the 10-fold cross-validation once instead of repeatedly as in Table 3. From Table 4 we can see that the optimal choice is the combination of all features, using a LogDemons smoothing parameter of σ = 1.5 and including dimensionality reduction using KPCA with a Gaussian kernel. Based on this table, we employed the optimal pipeline for different follow-up data, shown in Table 3, and compute a two-sample Hotelling's T-square test on the normalized SVFs.

**Table 4:** **Summary of the experimental results on baseline and 36 months follow-up images**.

**Feature**	**σ**	**KPCA**	**ACC**		**AUC**		**SEN**		**SPE**		**F1**	
			***L*^a^**	***G***	***L***	***G***	***L***	***G***	***L***	***G***	***L***	***G***
Baseline gray matter density	−	No	0.62		0.61		0.14		0.93		0.22	
Regional flux, all regions	−	No	0.66		0.57		0.16		0.98		0.27	
Regional flux, selected regions	−	No	0.72		0.64		0.44		0.90		0.55	
JD	1.5	Yes	0.74	0.73	0.73	0.32	0.63	0.14	0.81	0.96	0.65	0.23
Div	1.5	Yes	0.73	0.69	0.72	0.65	0.77	0.19	0.7	1.00	0.69	0.32
GL	1.5	Yes	0.67	0.63	0.66	0.50	0.63	0.30	0.70	0.94	0.60	0.43
SVF	1.5	Yes	0.76	0.66	0.80	0.64	0.70	0.86	0.81	0.58	0.70	0.69
Def	1.5	Yes	0.75	0.68	0.80	0.48	0.72	0.16	0.79	1.00	0.70	0.28
Combination	1.5	Yes	0.78	**0.92^b^**	0.79	**0.94**	0.74	**0.95**	0.81	0.90	0.73	**0.90**
Combination	0	Yes	0.62	0.87	0.45	0.91	0.02	0.91	1.00	0.85	0.04	0.85
Combination	3	Yes	0.76	0.85	0.69	0.85	0.67	0.93	0.81	0.79	0.68	0.82
Combination	6	Yes	0.66	0.86	0.62	0.89	0.37	0.77	0.84	0.93	0.46	0.82
Combination	1.5	No	0.68		0.69		0.65		0.70		0.61	

a *L means the linear kernel function, G means the Gaussian kernel function*.

b *Bold indicates the best performance*.

Considering a single feature, using the linear kernel, we furthermore see from Table 4 that the SVF and Def features perform the best, while the GL feature performs worst. The JD and Div features perform better than GL, but worse than SVF and Def. This may be related to the information content these features carry: SVF and Def encode the point-wise development as a vector field, JD and Div encode the local volume change but only as a scalar field, while GL only measures SVF amplitude, i.e., neighboring and directional information is missing.

## 5. Discussion

### 5.1. Analysis of the experimental results

In the smoothing parameter experiment, the worst performance for the SVF feature was obtained for σ = 0. Since in this case no regularization is performed, the resulting deformation may not be diffeomorphic and fails to represent real anatomical correspondence. The best performance is obtained for σ = 1.5. Too rigorous smoothing filters out the high frequency components in the SVF, and such details are important to distinguish between MCIc and MCIs subjects. σ = 1.5 seems a good compromise between regularization and the preservation of detail.

In the experiment that compares the classification performance of different features, the combination of all features outperforms any single feature in both ACC and AUC. By comparing different kernels, we find that the Gaussian kernel with the default parameter outperforms the linear kernel for the combination of all features.

From the ROC curves in Figure 5, we can see that for most of the features, KPCA dimensionality reduction largely improves classification performance. By introducing the max-margin mechanism, in theory the SVM classifier (Cortes and Vapnik, 1995) is able to deal with small sample sizes and high-dimensional features. However, in the real world, a small training set often fails to provide enough information to separate a high-dimensional feature space. As a result, dimensionality reduction is necessary for the SVM.

From the ROC curves in Figure 6, we can see that the normalization step can improve the classification performance. This result strongly supports the use of normalized features in classification.

When comparing the proposed method with two alternative methods on the study dataset, we can see that the proposed method outperforms them, as shown in Figure 8. Since the main difference between the MCIc and MCIs subjects is their different pathological and anatomical development, using only the baseline image does not provide such information. As a result, using the gray matter density of the baseline image performs worst among the three methods. The sparse regional flux feature also fails to obtain full developmental information, because it only measures the overall flux of the pre-defined structures and ignores many details. It is for example quite possible that two different developments have the same structural flux. Secondly, from the results in Table 4, dimensionality reduction using KPCA on the divergence feature slightly outperformed the method that aggregated divergence information over brain regions (the regional flux method). This may indicate that regional aggregation is not an optimal dimensionality reduction method, at least for this particular feature.

When comparing the results from follow-up data with different time intervals, we can see that even the short time interval (6 months for example) obtains a promising result. Previous results (Zhang and Shen, 2012; Liu et al., 2013) also showed that including short time interval follow-up data into the feature space can improve the results. As reported from Jack et al. (2013), brain structural changes may occur several years before Alzheimer's disease manifests itself. Similar to the sigmoid curve, the largest changes may happen at the mid-stage of the disease, rather then in the end stage. From Table 2, we can see that most MCIc patients have converted to “Dementia” or “MCI to Dementia” in the first 2 years. It is therefore possible that short follow-up times can still offer enough information for classification. This may indicate that the differences in development between the MCIc and MCIs groups appear even in the early stage of MCI, which is useful for early prediction of Alzheimer's disease.

### 5.2. Relation with methods from literature

In the last 5 years, predicting conversion from MCI to AD has received increasing attention, and many methods have been proposed based on structural MR images. The classification performance reported from some state-of-the-art methods are listed in Table 5. A direct comparison between these methods is difficult because of the different choice of cohort, preprocessing steps, validation strategy and reported measurement. However, from the reported performance and the method summary, some observations can be made.

**Table 5:** **Previous methods and results on classification of MCIs vs. MCIc**.

**Article**	**Data and feature**	**Feature type^a^**	**C / L^b^**	**Period (month)**	**N (MCIs,MCIc)**	**ACC (SEN/SPE)**
Chupin et al., 2009	Hippocampus and amygdalae segment	*R*	*C*	0–18	134, 76	64 (60 / 65)
Misra et al., 2009	Whole brain, ROIs VBM	*R*	*C*	0–36	76, 27	*82* (−/−)
Querbes et al., 2009	Cortex thickness	*R*, *DR*_*s*_	*C*	0–24	50, 72	73 (73 / 69)
Koikkalainen et al., 2011	Whole brain TBM	*V*	*C*	0–36	215, 164	72 (77 / 71)
Cuingnet et al., 2011	Hippocampus segment	*R*	*C*	0–18	134, 76	67 (62 / 69)
	Whole brain VBM(GM)	*V*	*C*			71 (57 / 78)
	Cortical thickness	*V*	*C*			70 (32 / 91)
Davatzikos et al., 2011	Whole brain VBM	*V*, *DR*_*s*_	*C*	0–36	170, 69	56 (95 / 38)
Westman et al., 2011	Cortical thickness and subcortical volumes	*R*	*C*	0–12	256, 62	59 (74 / 56)
Wolz et al., 2011	Hippocampus segment	*R*	*C*	0–48	238, 167	65 (63 / 67)
	Whole brain TBM	*V*	*C*			64 (65 / 62)
	Hip and amygdalae ROI, manifold learning	*R*, *DR*_*m*_	*C*			65 (64 / 66)
	Cortical thickness	*V*	*C*			56 (63 / 45)
	Combination of above					68 (67 / 69)
Cho et al., 2012	Cortical thickness	*V*, *DR*_*m*_	*C*	0–18	131, 72	71 (63 / 76)
Coupé et al., 2012	Hip and entorhinal cortex segment	*V*	*C*	0–48	238, 167	74 (74 / 74)
Zhang and Shen, 2012	Whole brain ROIs volume	*R*, *DR*_*s*_	*L*	0–24	50, 38	78 (79 / 78)
Eskildsen et al., 2013	Cortial ROIs TBM	*R*	*C*	0–48	227, 161	68 (68 / 69)
Liu et al., 2013	Whole brain ROIs volume	*R*, *DR*_*s*_	*L*	0–24	185, 164	71 (71 / 71)
Guerrero et al., 2014	Whole brain, manifold learning	*V*, *DR*_*m*_	*C*	0–24	114, 116	71 (75 / 67)
Lorenzi et al., 2015b	Whole brain regional flux	*R*	*L*	0–36	110, 86	65 (67 / 63)
Proposed method	SVF, Parallel Transport	*V*, *DR*_*m*_	*L*	0–36	43, 67	76 (70 / 81)
Proposed method	Combined, Parallel Transport	*V*, *DR*_*m*_	*L*	0–36	43, 67	*92* (95 / 90)

a *Feature Type: R means ROI-based feature; V means voxel or vertex based feature; DR_m_ means dimensionality reduction by feature mapping; DR_s_ means dimensionality reduction by feature selection. S means using single modality data, while M means using multi-modality data*.

b *L means longitudinal feature; C means cross-sectional feature*.

*Results are self-reported on different data*.

Several methods focus only on one or two brain structures that are believed to be directly related to the disease progression. Chupin et al. (2009) used the hippocampus and amygdala volume to obtain an accuracy of 64%. Coupé et al. (2012) focussed on the hippocampus and assigned an AD-likeness score to it. With the help of a better and more complex preprocessing pipeline, this method reported an accuracy of 74%. Wolz et al. (2011) and Cuingnet et al. (2011) reported accuracies of 67% and 65%, based on the hippocampus. These method use only cross-sectional information, and ignore the development over time.

Many methods use whole brain features in the MCI prediction task, either voxelwise or region-wise. Such whole brain features include Voxel Based Morphometry (VBM), Tensor Based Morphometry (TBM) and cortical thickness. Davatzikos et al. (2011) used cross-sectional VBM features to obtain an accuracy of 56%. Misra et al. (2009) used data-driven ROIs to extract ROIwise cross-sectional VBM features and reported a high accuracy of 82%. As mentioned in Guerrero et al. (2014), the ratio of positive and negative samples in this study is quite unbalanced, so that the reported result is hard to compare with other methods. Koikkalainen et al. (2011) used cross-sectional region-wise TBM on the whole brain to obtain an accuracy of 72%, while Eskildsen et al. (2013) focussed on cortical ROIs obtaining an accuracy of 68%. Several methods (Querbes et al., 2009; Westman et al., 2011; Wolz et al., 2011; Cho et al., 2012) used cross-sectional cortical thickness to predict MCI conversion. Querbes et al. (2009) used the cortical thickness within ROIs to obtain an accuracy of 73%. However, there is a bias in the ROI generation step as pointed out by Guerrero et al. (2014). Westman et al. (2011) used both subcortical structure volume and cortical thickness in predefined ROIs as features to achieve an accuracy of 59%. Cho et al. (2012) used a vertex wise cortical thickness to achieve an accuracy of 71%.

Beside these methods based on cross-sectional whole brain features, some methods extract longitudinal whole brain features. Liu et al. (2013) used the ratio of gray matter volume in baseline and follow-up images within 93 ROIs as features to obtains an accuracy of 71%. Zhang and Shen (2012) use longitudinal features from multi-modal data to train a multi-kernel SVM and achieved a good performance (accuracy of 78%). Lorenzi et al. (2015b) extracted the flux from the non-rigid registration between baseline and follow-up image of each structure as features.

From this summary we can see that both longitudinal as well as whole brain features are very powerful. Therefore, the proposed method is a meaningful choice since for the first time it exploits a *dense* whole brain feature (the SVF) that was derived from longitudinal information. Importantly, in our method the two visits are not treated independently as in Zhang and Shen (2012); Liu et al. (2013) but are related through registration. Moreover, our feature directly models anatomical change. The proposed method furthermore features a relatively straightforward skull stripping, and uses simple linear SVM classification and pre-processing steps.

### 5.3. Group-specific template selection

In the proposed method, a template image *T*_0_ is needed. Many VBM-based methods (Fan et al., 2007; Davatzikos et al., 2008, 2009, 2011; Fan et al., 2008b) use the MNI template during analysis. However, the MNI template maybe not a suitable choice for our application: (1) The MNI template is learned from a group of young and healthy subjects, while our study is on aged and MCI subjects; (2) The MNI template is averaged and blurred, potentially resulting in a sub-optimal registration, losing details. The results from Section 4.3, furthermore support the use of a template selected from the MCI population.

### 5.4. Future work

Although our method obtains a good classification result, there are still some points that can be improved. For the anatomical correspondence estimation, the proposed method uses only one follow-up image. State-of-the-art image regression methods (Singh et al., 2015; Sun et al., 2015) compute the developmental trajectory from multiple follow-up images, which may help to estimate an improved correspondence. For the dimensionality reduction, we only use KPCA. Better dimensionality reduction methods, such as t-distributed Stochastic Neighbor Embeddings (t-SNE) (Van der Maaten and Hinton, 2008) may help to increase the classification performance. This study uses a relative small dataset, and a larger dataset may help the classifier to learn a better model. Including more types of features, like gray matter density, cortical thickness or structure volume, may also improve the result, as different types of features can represent different properties of the brain.

## 6. Conclusion

In this paper, we propose a new method to detect MCI conversion using the combination of normalized features from longitudinal structural MR images, with KPCA dimensionality reduction. Using the proposed method, we obtain a high classification performance. The alignment of SVFs in the template space enables a statistical comparison between the MCIc and MCIs group on a voxel level. This facilitates the inspection of differences, which were found in the hippocampus and the ventricles.

We conclude that the estimation of structural change in the brain over time can aid in predicting if MCI will evolve to Alzheimer's disease. Such finding may be useful for early identification of AD patients, to delay the disease onset or to slow down the disease progression. Therefore, it may be useful to improve the quality of life of the patient and decrease the societal cost related to AD.

## Author contributions

ZS, MV, BL, and MS contributed to the designing the work, drafting, final approval and all aspects of this work.

### Conflict of interest statement

The authors declare that the research was conducted in the absence of any commercial or financial relationships that could be construed as a potential conflict of interest.

## Footnotes

^1^The latest version of this file was updated in 2012, so it only contains part of the data of the current ADNI dateset.
